# The Environmental Impact of a Wave Dragon Array Operating in the Black Sea

**DOI:** 10.1155/2013/498013

**Published:** 2013-06-06

**Authors:** Sorin Diaconu, Eugen Rusu

**Affiliations:** Department of Applied Mechanics, University Dunarea de Jos, 800201 Galati, Romania

## Abstract

The present work describes a study related to the influence on the shoreline dynamics of a wave farm consisting of Wave Dragon devices operating in the western side of the Black Sea. Based on historical data analysis of the wave climate, the most relevant environmental conditions that could occur were defined, and for these cases, simulations with SWAN spectral phase averaged wave model were performed. Two situations were considered for the most representative patterns: model simulations without any wave energy converter and simulations considering a wave farm consisting of six Wave Dragon devices. Comparisons of the wave model outputs have been carried out in both geographical and spectral spaces. The results show that although a significant influence appears near the wave farm, this gradually decreases to the coast line level. In order to evaluate the influence of the wave farm on the longshore currents, a nearshore circulation modeling system was used. In relative terms, the longshore current velocities appear to be more sensitive to the presence of the wave farm than the significant wave height. Finally, the possible impact on the marine flora and fauna specific to the target area was also considered and discussed.

## 1. Introduction 

The higher request concerning the implementation on large scale of the renewable energy imposed by the EU directives also implies a substantial enhancement of the renewable energy extraction all over Europe. 

Wave energy is abundant and is more predictable than wind or solar energy. Although the amount of energy that can be extracted using wave technologies varies depending on the location and weather conditions, wave energy can be accurately predicted using numerical models within a window of a few days. Wave energy also offers much higher energy densities, allowing devices to extract more power from a smaller volume at consequently lower costs.

Shoreline energy converters have been tested for some years, and several successful devices have been installed. Nevertheless, the most exciting developments at the present time are in extracting renewable energy in the nearshore and offshore areas.

Combined wind-wave projects, also known as hybrids, hold great potential down the line when wave technologies become more established. At that point, wave production might compensate for the intermittency of the offshore wind, while economies of scale developed from offshore wind could accelerate cost reduction for wave components. Although nowadays discussion of hybrid offshore wind-wave projects is limited more to demonstrations or pilot projects, it is expected that in the near future the synergy between wave and wind energy would be better achieved and hybrid platforms will become fully operational and economically sustainable. Despite a certain degree of uncertainty related to the variability in the wave-wind climate, improvements in the accuracy of evaluating the environmental data in the coastal areas would enhance also the accuracy of the predictions that future energy convertors yield. Some economic advantages of combining the wave and wind power productions are presented in [1, 2].

The target of the present work is a coastal area located on the western side of the Black Sea, which is not considered an environment rich in wave energy. On the other hand, due to the technological developments regarding harvesting renewable energy resources, which are expected to be very high in the near future, this area can become interesting especially in relationship with the hybrid projects combining the marine energy from waves, wind, marine currents, thermal gradients, and differences in salinity.

Until now, several evaluations of the wave conditions and of the wave energy resources in the Black Sea have been made, and among these, the most relevant are those of presented in [3–6], where the presence of various hot spots from the point of view of the wave energy has been identified. These hot spots are areas near the coast where significant differences in terms of wave conditions usually appear.

Harvesting the wave energy and transform it into electricity implies wave energy convertors (WECs) that transform in the first stage the wave energy into mechanical energy, and then this is again transformed into electricity. Several types of devices as well as an overview on the WEC evolution are given in [7]. Sea waves generate high forces at low velocities, and the hydraulic systems seem to be the most appropriate devices to absorb the energy in such conditions. The device is fixed at a location with a mooring system. Electricity is transmitted to the sea bottom through a flexible cable and afterwards to the coast by a cable line. The waves depend on the characteristics of the wind that generates them, and in general the energetic conditions are significantly higher in the wintertime than in summertime. Both power production and cost are dependent on the layout of the farm. To develop a commercial technology, the impact of arranging WECs in a farm has to be investigated as well. An optimization of such a wave energy farm operating in the North Sea is presented in [8]. 

On the other hand, the implementation of the energy farms is depends of a correct evaluation of their impact on the coastline dynamics, because changes might appear in relationship with the energy and the direction of the waves as they propagate from the energy farm further towards the coast. The environmental impacts of the wave energy farms are yet insufficiently studied. Although this impact should not be expected as necessarily negative, since reducing the wave energy might produce benefits in several coastal areas, evaluating the sensitivity of the nearshore wave climate to the extraction of the renewable energy still represents a very important issue, and a lot of studies are required in this direction.

In this context, the objective of the present work is to evaluate the coastal impact of a WEC array composed of six Wave Dragon devices disposed in one line that would operate on the west side of the Black Sea.

Nørgaard et al. [9, 10] showed the importance of such devices, which can be used also to reduce the wave height along the shorelines. Different stiffness of the mooring system and reflector joints have been tested for different wave steepness and relative floating ratios assessing the influence of each of these parameters on the wave transmission. 

Some other studies are those of Millar et al. [11] for the Wave Hub project or by Palha et al. [12] that studied the effect of a Pelamis wave farm on the shoreline wave climate which is situated close to the Portuguese coast and also by Ponce de Leon et al. [13] that studied the influence of a wind farm in the nearshore. The impact on the coastal dynamics is dependent both on the bathymetric features and on the particularities of the environmental matrix. For this reason, extended evaluations should be carried out in each coastal environment where a new structure or the energy farm will be installed. These factors affect the medium and long-term changes induced in the shoreline wave climate and dynamics. 

From this perspective, the present study might represent a step forward to the investigation of the potential impact of the implementation of large-scale wave energy arrays by providing some insight in relationship with the influence of a Wave Dragon-based farm that would operate in the coastal environment. The present target area is located in the western side of the Black Sea close to the mouths of the Danube River, and this was found to be one of the most energetic parts of the western side of the sea [14]. Moreover, the results of the present work can be easily extrapolated to many other coastal environments.

## 2. Theoretical Background of the Numerical Models Considered

Since a deterministic approach of the sea waves is in general not feasible, the most adequate representation of the waves is based on the spectral concept. The wave spectrum represents the Fourier transform of the autocorrelation function of the free surface elevation. The spectral wave model considered in the present study is Simulating Waves Nearshore (SWAN, [15]). This is considered the state-of-the-art phase averaged shallow water wave model and solves the wave action density balance equation which can be expressed as

(1) ∂∂tN+∂∂xCgxN+∂∂yCgyN  +∂∂σCσN+∂∂θCθN=Sσ,

where *N* is the wave action density and *C*_*gx*_, *C*_*gy*_, *C*_*σ*_, and *C*_*θ*_ represent the propagation speeds in the geographical space (*x*, *y*), in the frequency space (*σ*), and in the directional space (*θ*), respectively. *S*/*σ* represents the source and sink terms that account in deep water for processes as wave generation by wind, whitecapping dissipation, and nonlinear wave-wave interactions (quadruplets). In shallow water, additional processes as bottom friction, depth-induced breaking, and triad wave-wave interactions are also introduced. The model can be now utilized with either Cartesian or spherical coordinates; it has a parameterization to counteract the garden sprinkler effect, which is a characteristic of large areas and also includes a phase-decoupled diffraction approximation. 

Many phenomena are generated from the wave energy dissipation in the surf zone by breaking, but for a practical application, the generation of the longshore currents is the most significant, obtaining considerable strength and being a significant factor in controlling the morphology of the beaches. They can also have an impact on human activities in the coastal zone. Calculation of the current velocity is usually based on radiation stress theory (Longuet-Higgins [16]), and various 1D, 2D, and 3D numerical models have been developed to predict these currents. A widely known general prediction system for nearshore circulation is SHORECIRC (Svendsen [17]). This is a quasi-3D model that combines a numerical solution for the depth-integrated 2D horizontal momentum balance equations with an analytical solution for the 3D current profiles. The restrictions of the model are very mild, and the basic circulation equations solved can, therefore, in general be considered very accurate. In addition, such a model catches the nonlinear feedback between wave-generated currents and the waves that generate them. Nevertheless, the model works in the time domain and is quite expensive in terms of computational resources. A simpler, but considerably faster, model is Surf, or Navy Standard Surf Model (NSSM), [18]. This is a parametric one-dimensional model that estimates the wave-induced longshore currents by solving the following equation for the longshore current:

(2) $$\begin{array}{c} \tau_{y}^{r}+\rho \frac{\partial }{\partial x}\left[\mu h\frac{\partial V}{\partial x}\right]-\langle \tau_{y}^{b}\rangle +\tau_{y}^{w}=0. \end{array}$$

The first term in this equation, *τ*_*y*_^*r*^, represents the longshore directed radiation stress due to the incident waves, the second term represents the horizontal mixing term due to cross-shore gradients in the longshore current velocity *V*, the third term, *τ*_*y*_^*b*^, is the wave-averaged bottom stress, and the last term, *τ*_*y*_^*w*^, represents the longshore wind stress. The model includes a parametric relation for cross-shore growth, and dissipation of waves due to breaking and additional relations are included for estimating percent breaking, the number of lines of breakers, and breaker type. Because NSSM is one-dimensional several assumptions are utilized. In particular, the bottom contours are considered straight and parallel, the current depth uniform, and directional wave spectra narrow banded in frequency and direction.

Evaluations in the Italian nearshore of the waves and nearshore currents were performed by Conley and Rusu [19] with SWAN and NSSM models, and their results proved that this approach can be considered reliable for a wide range of coastal applications. In order to increase the properties of the two models and for simplicity and reliability, Rusu et al. [20] joined the two models in a user friendly computational tool named as the “Interface for SWAN and Surf Models” (ISSM). 

The computational domain is illustrated in Figure 5. This is a rectangle with about 17.5 km in *x-*direction (cross shore) and 20 km in *y*-direction (long shore). The main characteristics and physical processes activated are presented in Table 1. In this table, Δ*x* and Δ*y* represent the resolution in the geographical space, Δ*θ* is the resolution in the directional space, *n*_*f*_ is the number of frequencies in the spectral space, *n*_*θ*_ is the number of directions in the spectral space, *n*_*gx*_ is the number of the grid points in *x*-direction, *n*_*gy*_ is the number of grid points in *y*-direction, and *n*_*p*_ is the total number of grid points. 

Some details will be given next in relationship with the implementation of the modeling conditions in the target area. The input fields considered are also indicated in Table 1 as follows: *wave* represents the wave forcing, *tide* is the tide forcing, *wind* represents the wind forcing, and *crt* is the current field. The physical processes activated are coded as follows: *gen* is the generation by wind, *wcap* indicates the whitecapping process, *quad* represents the quadruplet nonlinear interactions, *triad* indicates the activation of the triad nonlinear interactions, *diff* is the diffraction process (phase decoupled), *bfric* represents the bottom friction, *setup* is the wave-induced setup, and *br* indicates the activation of the depth-induced wave breaking.

## 3. Main Particularities of the WEC and of the Wave Conditions in the Target Area

The WEC considered in the present work is the Wave Dragon (Kofoed [21]). The basic idea of this wave energy converter device is to use well-known and well-proven principles of traditional hydropower plants in an offshore floating platform of the overtopping type.

The device elevates waves to a reservoir where water is passed through a number of turbines and in this way transformed into electricity. This is a typical terminator type WEC, for which the conservative approach is to assume that the devices will absorb all suitable wave energy across the full width of the reservoir. 

The Wave Dragon (Figure 4) consists of two wave reflectors that direct the waves towards a curved ramp which overtops in a water reservoir and, therefore, has an increased potential energy compared to the surrounding sea. Thus, the Wave Dragon directly utilizes the energy of the water's motion.

To reduce rolling and keep the platform stable, the Wave Dragon must be large and heavy, having only one kind of *moving parts*: the turbines. This makes it to be a durable and resistant structure. This is essential for any device bound for operations offshore, where extreme conditions and fouling seriously affect any moving parts. If the waves do not interact with the ramp, they are reflected under its structure or diffracted away. Also, to improve the device performances, two reflectors are placed and hinged to the platform, which reflect the waves towards the ramp. The experiments showed that the ramp must be short to reduce the loss of energy, and due, the elliptical form to the overtopping increases significantly. 

Some remarks on the wave energy potential of the Black Sea near the Romanian coasts together with a possible power take off system that can be placed here are given in [22]. Onea [23] made an estimation of the expected power provided by some wave energy devices operating in the western side of the Black Sea. This was based on the analysis of the wave data registered at the Gloria drilling unit for a five-year period (2001–2005). Considering the above data, diagrams for the bivariate distributions of the sea states occurrences, defined by the significant wave height and the energy period, were designed for both winter and total time. On this basis, the efficiency of different technologies for the extraction of the wave energy, including the Wave Dragon, was assessed. The above results showed that a Wave Dragon device would produce close to the target area about 600 kW electric power in winter time and about 400 kW for total time, respectively.

The device has a very complex design because there must be a perfect relationship between ramp, wave reflectors, wave height, the floating height of the device, and the amount of water overtopped and stored in the reservoir (Figure 4(b)). The components are all well-established technologies, and the Wave Dragon is a particular application combining these to produce electricity from the waves.

**Figure 1:**
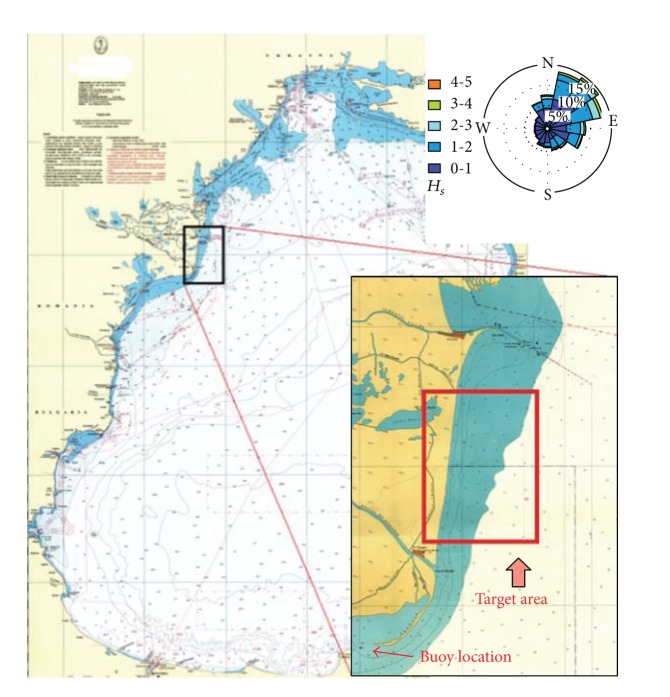
Location of the target area and the wave conditions resulting from an analysis of 5 years of data (2006–2011).

The target area considered in the present study is found to be among the most energetic sites from the western side of the Black Sea and is located at the south of Sulina channel, which is also a very important navigation sector since it represents the main gate in the seventh Trans-European transportation corridor (Figure 1). It has to be highlighted also that in this region the wave fields are characterized by significant variations during the year.

The Romanian Black Sea littoral evolution of the sea-land interface, for a period of several decades, had registered some significant variation. Mateescu et al. [24] presented a study of the beach short-term response under the action of the marine factors in the actual geomorphologic conditions. The previously stated results indicate a significant coastal response process to the climate changes/sea level rise trend, with an obvious influence on the future development of the natural environment, as well as on the socioeconomic activities in coastal space. A study to determine various geomorphic types of landforms in order to create a web geomorphic classification development for the Black Sea coast has been made by Stanica et al. [25]. 

**Figure 2:**
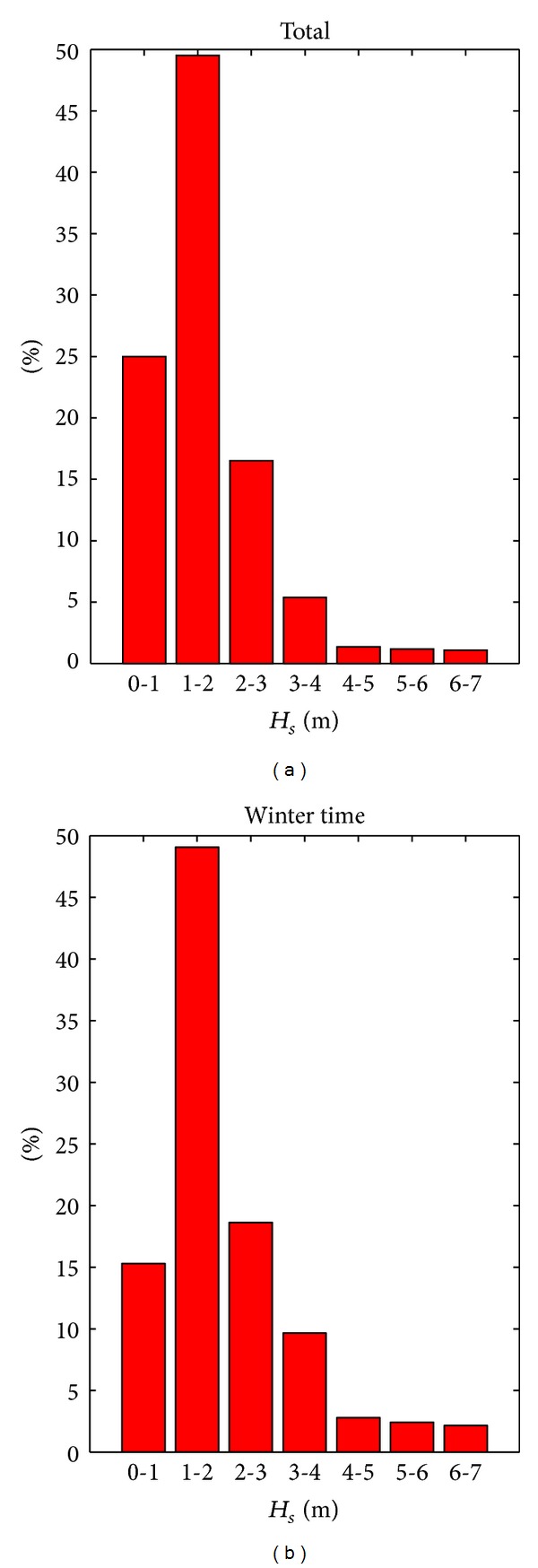
Analysis of the wave data measured at buoy close to the target area in the period 2006–2011: (a) Classes of significant wave height (*H*_*s*_) for the total time interval; (b) *H*_*s*_ classes for wintertime.

**Figure 3:**
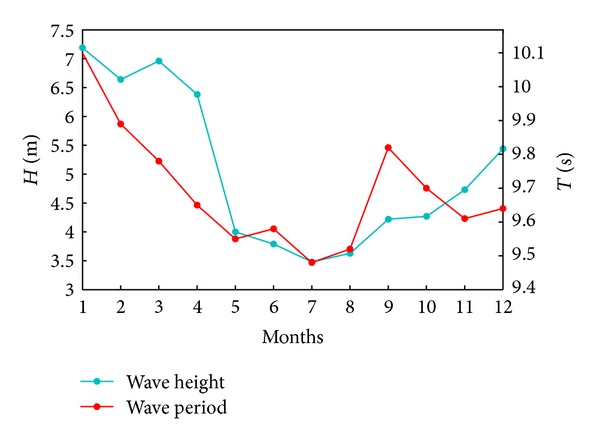
Analysis of the wave data measured at a buoy close to the target area in the period 2006–2011: *H* (m) monthly maximum wave height; *T* (s) monthly maximum wave period.

**Figure 4:**
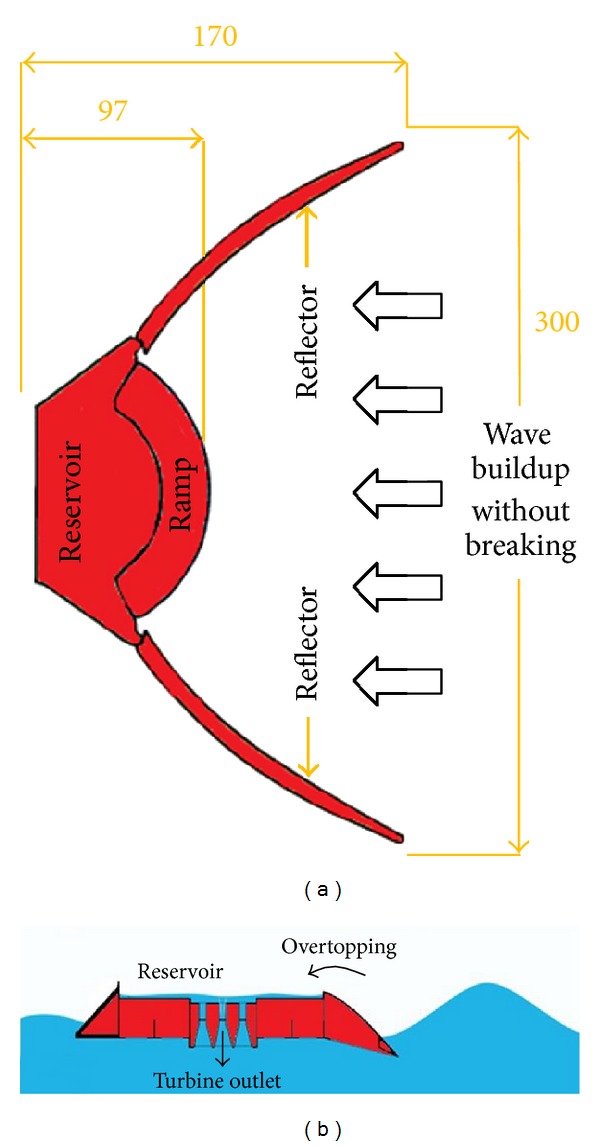
(a) Main structural elements of a Wave Dragon WEC in plain view—dimensions in meters; (b) cross-sectional view of the reservoir part of the Wave Dragon.

The wave data analysis presented in this section considers data measured at a buoy which operated in the western sector of the Black Sea close to the target area (Figure 1). The measurements were made daily in the five-year time interval 2006 and 2011. The results were structured for total and winter time, respectively. In this work, winter time represents the time interval between October and March. Figure 1 shows together with the target area the directional distributions of the *H*_*s*_ classes as reflected by the buoy measurements. It can be observed that the lowest wave heights correspond to the western direction because of the presence of the coast in that side while the dominant wave direction is from the northeastern side. It can be also seen that from the same direction higher waves are usually coming in comparison with other directions. In Figure 2, the *H*_*s*_ classes are presented in percents in terms of the number of occurrences, illustrating in parallel the results for total time (a) and wintertime (b), respectively. The monthly maximum values of the significant wave heights and mean wave periods are shown in Figure 3.

The results show that the highest probability of occurring waves with significant heights, greater than 7 m is in the time interval between December and January. This possibility begins in September and lasts until the end of March. The same evolution can be seen for the significant wave heights in the classes 4-5 m, 5-6 m, and 6-7 m. Waves with significant wave heights in the range 1-2 m are present in a considerable proportion all over the year, with a minimum in March and a maximum in July. For the waves smaller than 1 m, the frequency of occurrence in summertime is almost double than in wintertime. The highest value of the significant wave is 7.08 m and corresponds to waves coming from the northeastern direction. As regards the wave periods, there are not so relevant differences between winter and total time. 

## 4. The Expected Impact of the Wave Dragon Farm on the Marine Vegetation and Fauna That Characterize the Target Area

An important issue concerning the deployment and exploitation of the future energy farms relates to a correct assessment of their environmental impact, in general, and of their impact on the aquatic flora and fauna, in special. 

It is thus very important to have a comprehensive picture of all the physical and biological characteristics of the area targeted in order to be able to assess correctly the consequences of the wave energy extraction. From this perspective, [26] studied the main physical-chemical characteristics correlated with the biological specificity of different species of multicellular algae along the Romanian Black Sea coast while [27] presented the evaluation of the conformity level for the marine environment of the Romanian marine areas designated for the main molluscs growth and exploitation. 

The soils in the Black Sea basin are varied, and their distribution reflects the connection with the principal genetic factors (lithology, relief, climate, vegetation, and fauna) and the influence of the human activities by modifying the local conditions. From the same perspective, Stanica et al. [28] revealed the aspects that affected the natural processes of the Black Sea coast near the Sulina mouth by the human activities leading to erosion of the coast.

Previous studies have found a higher proportion of plant species along the coastal area of the Black Sea. Anastasiu et al. [29] assessed the role of the harbours as gateways and reservoirs for alien plant species, the structure and invasion pattern of the alien plants, and test methods useful for effective monitoring programs; on the other hand Sava et al. [30] showed the influence of the nutrients on the macrophytic red algae of the Romanian Black Sea coast.

An overview of the turbot *Psetta maeotica* species that populate the Romanian Black Sea and the importance of the regional fishing potential under the aspect of market demand, both on the national and international level, is made in [31], while in [32] a study of the distributional patterns of the zoobenthos from the artificial hard substratum is presented.

Thus, in the global environmental context, it is assumed that the presence of a Wave Dragon farm would have a positive impact as an alternative to the use of polluting fossil fuels for generating electricity. These devices are a clean power generation technology with many environmental advantages: they have a very low visibility (Wave Dragon can be compared to a moored ship and will have a maximum height above mean sea level of 7 meters), the underwater noise generation is very low (so it cannot produce harm to the marine fauna due to noise), they have a modest “footprint” on the seabed from anchor block and the power cable duct, and there is no risk of spill (they use water hydraulics, and no toxic antifouling is used). 

From this perspective, the impact of a single WEC on the marine environment is expected to be small, but the presence of a large number of converters in the same area working in an almost continuous way may cause eventually some environmental impact. Of course, this impact can be significantly attenuated: subsea cables and onshore cables impact can be avoided by identifying the important habitats for fisheries, benthos, and so forth and avoiding laying cables in these areas. Locations have to be chosen with respect to commercial and recreational fisheries, but we can notice positive effects on fish resources (this area will create a fishery exclusion zone, and the artificial reef effect will attract fish).

Nevertheless, limited studies have been done regarding the wave energy farms impact and changes that these devices can make on the waves and current field. Wave Dragon farms will extract energy from waves and do some extended changes of the hydrodynamics behind the farm. Wave heights are expected to decrease behind a Wave Dragon farm. Changes in the hydrophysical regime due to the extraction of energy from the waves may cause an impact on coastal processes as erosion and sediment transport and a reduced recreational value, regarding surfing due to smaller waves. Therefore, the waves and current estimations are important aspects that must to be taken into account, and these aspects will be evaluated and discussed in the next section. 

## 5. Model System Simulations and Discussion of the Results

As in the case of the attenuator type devices, the efficiency of the terminator devices is directionally dependent; that is, they must follow the direction of the wave propagation. Simulations with the SWAN model have been performed in various cases that reflect better the most relevant wave patterns in the target area. 

For accounting in the wave model of the Wave Dragon array geometry, the command obstacle that is available in SWAN was considered. The obstacle is subgrid in the sense that it is narrow compared to the spatial meshes, but its length should be at least one-mesh long. The location of the obstacle is defined by a sequence of corner points of a line. The obstacles interrupt the propagation of the waves from one grid point to the next. Such an obstacle will affect the wave field in three ways: it will reduce the wave height of waves propagating through or over the obstacle all along its length, it will cause waves to be reflected, and it will cause diffraction around its end. Therefore, the model can reasonably account for waves around an obstacle if the directional spectrum of incoming waves is not too narrow. There are several mechanisms for transmission of waves. In SWAN, this can be computed as transmission of waves passing over a dam with a closed surface or as a constant transmission coefficient which was the choice in the present work. Together with the command obstacle, either specular reflection, when the angle of reflection equals the angle of incidence, or diffuse reflection, where incident waves are scattered over reflected direction, may be considered. In this way, the effect on the waves in front of the wave arrays might be also accounted for. To accommodate diffraction in SWAN simulations, a phase-decoupled refraction-diffraction approximation is implemented. It is expressed in terms of the directional turning rate of the individual wave components in the 2D wave spectrum. The approximation is based on the mild-slope equation for refraction and diffraction, omitting phase information. Therefore, this does not permit coherent wave fields in the computational domain. According to the technical data of the Wave Dragon device the transmission coefficient was set to 0.68 and the diffuse reflection coefficient to 0.2 (according to Harrington [33]).

### 5.1. Evaluations in the Geographical and in the Spectral Spaces

An in depth analysis of the wave conditions was performed. These correspond to two different situations that were considered in the present study, WD0 (without any device operating in the target area) and WD6 (with six Wave Dragon devices operating in line in the target area). 

**Figure 5:**
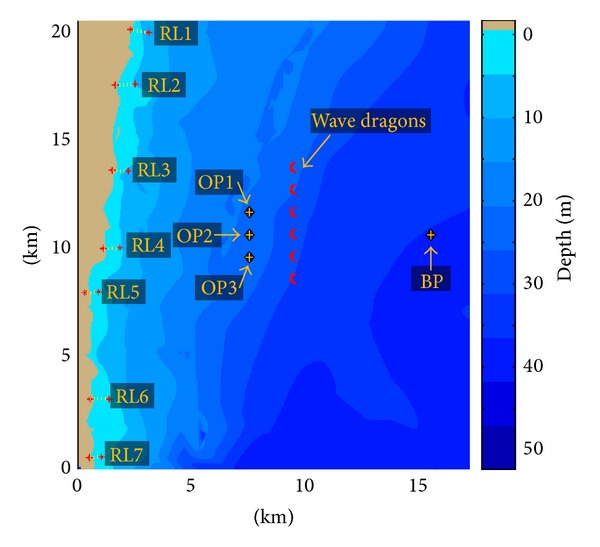
The computational domain considered for the simulations with numerical models. In the background the bathymetry is represented while in the foreground the Wave Dragon, the reference points, and the reference lines. BP indicates the boundary point, OP are the offshore points, and RL represent the reference lines considered in the analysis of the nearshore currents. Each offshore extremity point of the above reference lines is denoted as NP (nearshore point).

In Figure 5, some reference points are illustrated; the first reference point is denoted as BP and indicates the boundary point, and three other reference points are defined at 1.8 km down wave from the WD farm, and they have been denoted as offshore points (OP). Moreover, in order to assess the coastal impact of the wave farm by evaluating the wave-induced nearshore currents, seven reference lines (RL) were positioned along the entire coast and they are denoted as RL1 to RL7. The extremities of each reference line from the offshore side denoted as nearshore points (NP), and these points were taken into consideration for analyzing in both geographical and spectral spaces the nearshore waves.

**Figure 6:**
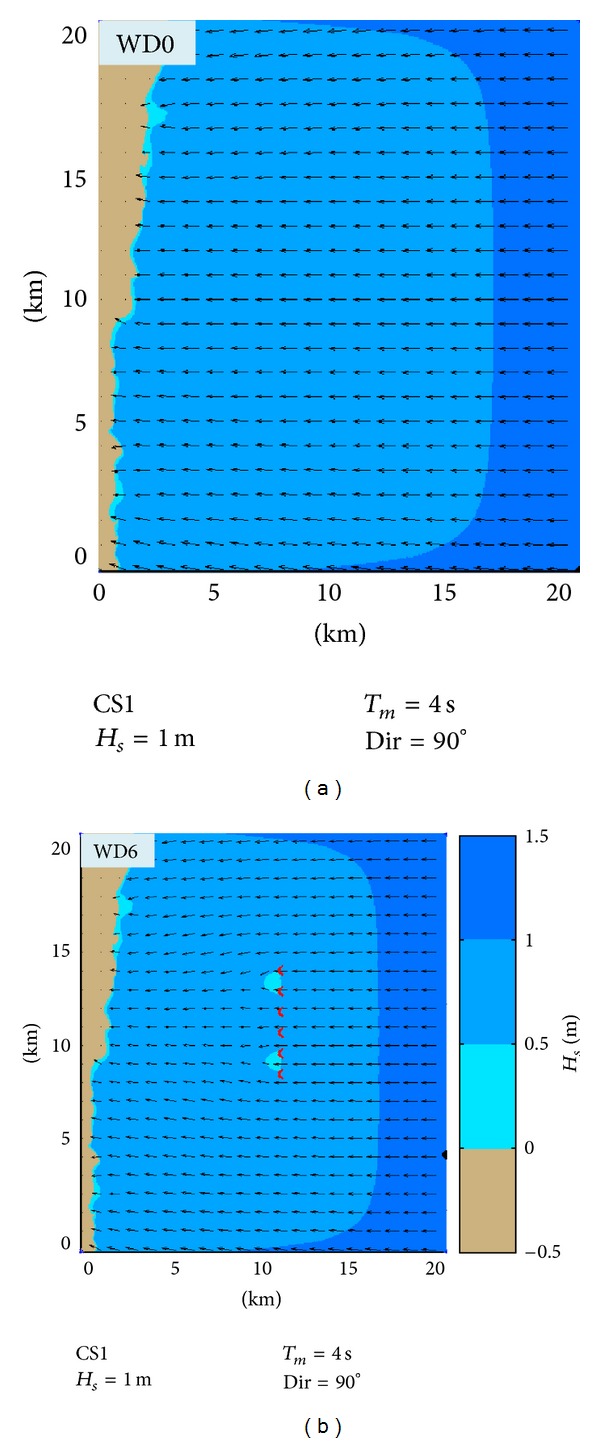
Evaluation in the geographical space of the impact on the wave field of a wave farm based on Wave Dragon WECs that operate in the target area. CS1—average to high energetic conditions and waves coming from east (90° in nautical convention). (a) SWAN simulation in the case without Wave Dragons (WD0). (b) SWAN simulation in the case when six Wave Dragons operate in line (WD6). The *H*_*s*_ scalar fields are presented in the background while in the foreground the wave vectors are indicated.

**Figure 7:**
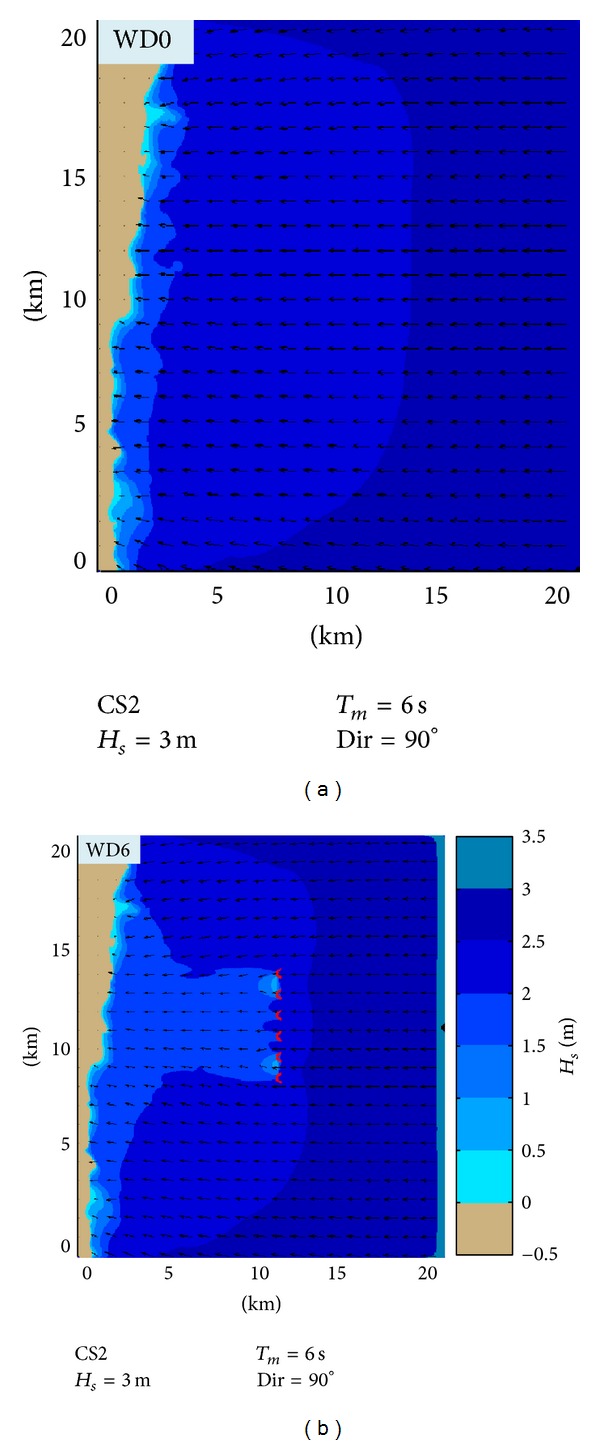
Evaluation in the geographical space of the impact on the wave field of a wave farm based on Wave Dragon WECs that operate in the target area. CS2—high energetic conditions and waves coming from east (90° in nautical convention). (a) SWAN simulation in the case WD0. (b) SWAN simulation for the case WD6. The *H*_*s*_ scalar fields are presented in the background while in the foreground the wave vectors are indicated.

In Figures 6 and 7, the impact in the geographical space on the wave field of a wave farm based on Wave Dragon devices for two different case studies is presented: CS1 (*H*_*s*_ = 1 m, *T*_*m*_ = 3 s, and Dir = 90°) and CS2 (*H*_*s*_ = 3 m, *T*_*m*_ = 6 s, and Dir = 90°). 

These cases were chosen because it has been observed that they present the highest differences between the two situations: with and without the energy farm. Thus, at the same time the two situations which were considered are presented in the figure, without any device deployed in the target area (WD0) and when six Wave Dragon devices operate in line (WD6), respectively.

CS1 corresponds to average wave conditions and Figures 6 and 7 show that in this case the impact is only locally visible, the wave field being attenuated on a small area down wave the farm. Nevertheless, as the wave height increases, the impact propagates further towards the coast, like in CS2.

**Figure 8:**
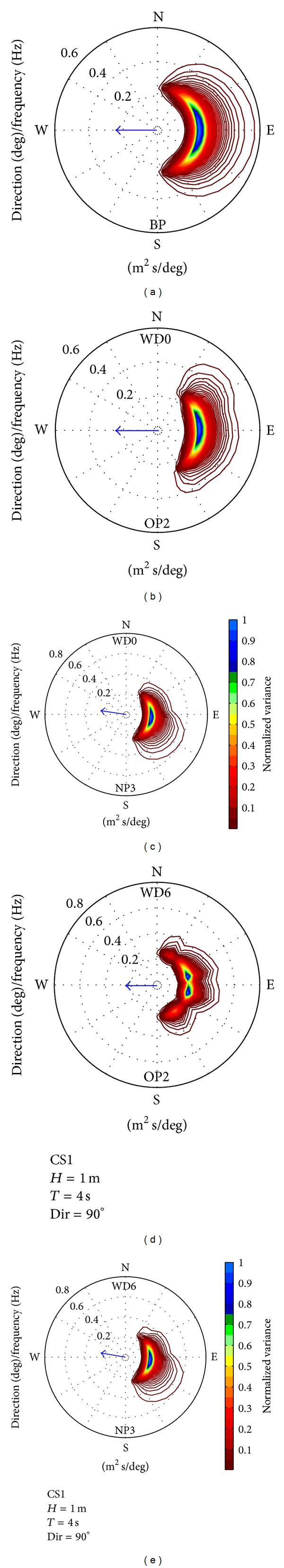
Evaluation in the spectral space of the impact on the wave field of a wave farm based on Wave Dragon WECs that operate in the target area for CS1. (a) BP for WD0. (b) OP2 for WD0. (c) NP3 for WD0. (d) OP2 for WD6. (e) NP3 for WD6.

**Figure 9:**
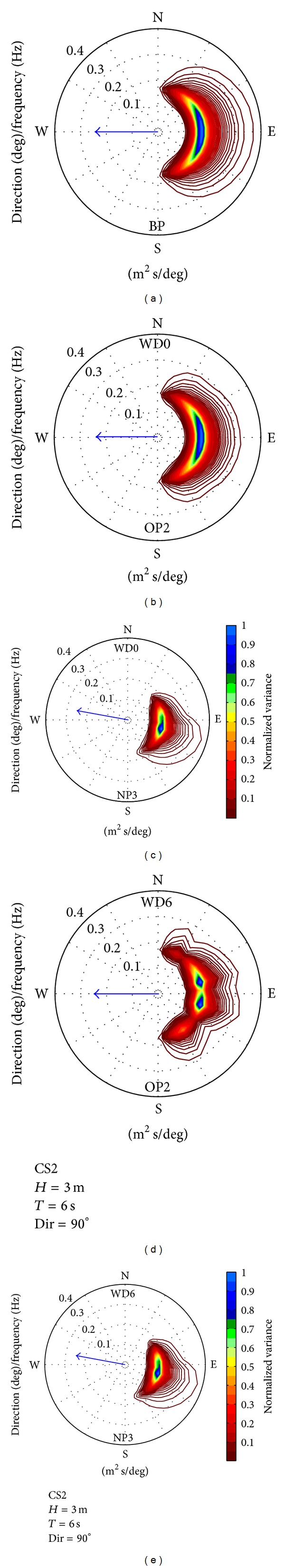
Evaluation in the spectral space of the impact on the wave field of a wave farm based on Wave Dragon WECs that operate in the target area for CS2. (a) BP for WD0. (b) OP2 for WD0. (c) NP3 for WD0. (d) OP2 for WD6. (e) NP3 for WD6.

 The evaluation in the spectral space of the Wave Dragon energy farm impact is illustrated in Figures 8 and 9 for the same two case studies (CS1 and CS2), where the 2D wave spectra were analyzed in parallel in the reference points OP2 and NP3 for the two different configurations considered (WD0 and WD6). In this figure a JONSWAP type spectrum was considered.

The boundary point (BP) presents the wave conditions unaffected in any way by the presence of the wave farm. Due to the presence of the Wave Dragons, the single-peak JONSWAP spectrum is transformed in a double peak spectrum immediately after the WEC array (as, e.g., in OP2), but this spectral shape does not propagate further in the geographical space, and at the level of the nearshore (the reference point NP3) no significant difference occurs in terms of the spectral shapes between the two different configurations considered (WD0 and WD6).

In Tables 2 and 3, a detailed data representation of the wave variation is given for CS1 and CS2, respectively. This represents the values of the wave parameters in all the reference points defined (BP, OP1, OP2, OP3, NP1, NP2, NP3, NP4, NP5, NP6, and NP7) for the two configurations considered (WD0 and WD6). 

Some other relevant situations are presented in Tables 4, 5, and 6; this time the analysis is being focused only on the offshore points (OP1, OP2, and OP3) where the influence of the wave energy farm is in fact really relevant for the two situations mentioned before. The parameters considered in Tables 2, 3, 4, 5, and 6 are significant wave height (*H*_*s*_), maximum variance (*E*_max⁡_), mean wave direction (Dir), directional spreading (DSPR), peak period (*T*_*p*_), mean period (*T*_*m*_), wavelength (Wlen), the components of the energy transport (*P*_*x*_, *P*_*y*_), and the components of the wave forces (*F*_*x*_, *F*_*y*_). 

The results presented in the above tables show again that indeed relevant differences occur at the offshore reference points that were defined, while as regards the nearshore point NP1–NP7, these differences are significant attenuated. 

### 5.2. Assessment of the Impact on the Shoreline Dynamics

Various phenomena are generated by the energy dissipation in the coastal environment, and the most relevant are the nearshore currents because they contribute to the sediment transport affecting directly the coastal dynamics. It is thus very important to find out how an energy farm will affect the nearshore circulation patterns by its presence in the marine environment and to estimate which will be the medium to the long-term impact on the coastal dynamics of the energy farm.

The nearshore currents were evaluated along the reference lines RL1–RL7, for the two different configurations considered (WD0 and WD6). The results concerning the maximum longshore current velocity are presented in Table 7. Table 7 presents the results corresponding to *H*_*s*_ = 1 m, *H*_*s*_ = 3 m, and *H*_*s*_ = 5 m at three different wave directions (30°, 90°, and 150°).

**Figure 10:**
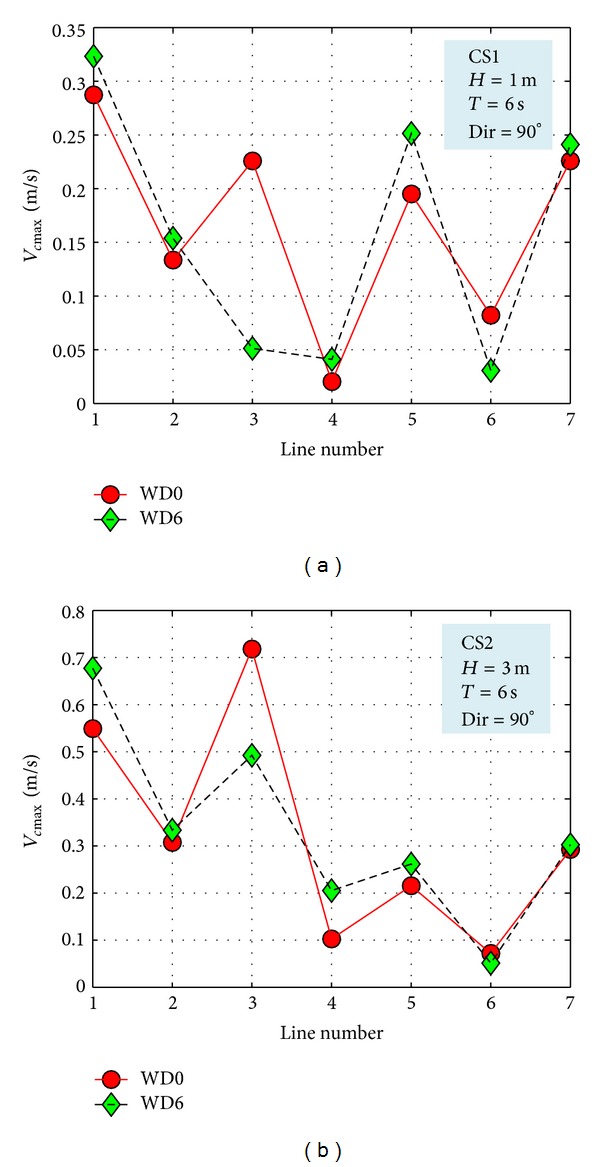
Evaluation of the impact of the energy farms on the maximum velocities of the nearshore currents along the reference lines considered. (a) CS1, (b) CS2.

The maximum values of the velocities of the nearshore currents along the reference lines are illustrated in Figure 10 for both case studies considered (CS1 and CS2). As the results show, the influence of the wave farm over the nearshore currents appear in all the points but in general is not very high. From the analysis of data from the simulations, it has been observed that the most sensitive direction is that normal to the shoreline (90°) and the highest decrease of the current velocity appears in NP3. 

**Figure 11:**
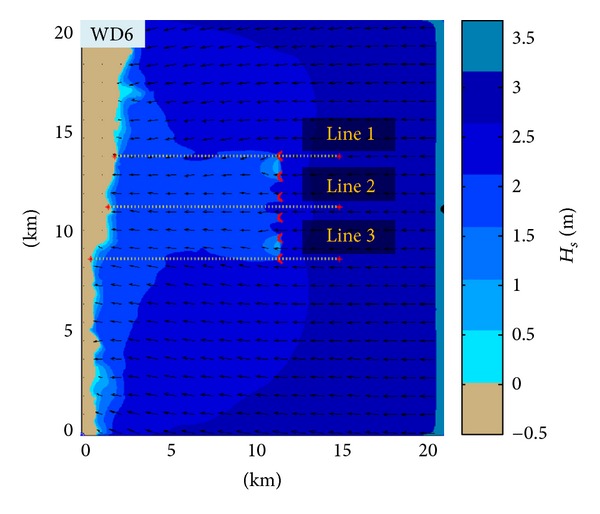
Evaluation of the impact of the energy farms on the maximum velocities of the nearshore currents along the reference lines considered. (a) CS1, (b) CS2.

An additional issue is related to the assessment of the evolution of the waves after their impact with the body of the WD farm structures. For that, the *H*_*s*_ variations have been analyzed along three reference lines passing through the wave energy farm in different locations, as illustrated in Figure 11.

**Figure 12:**
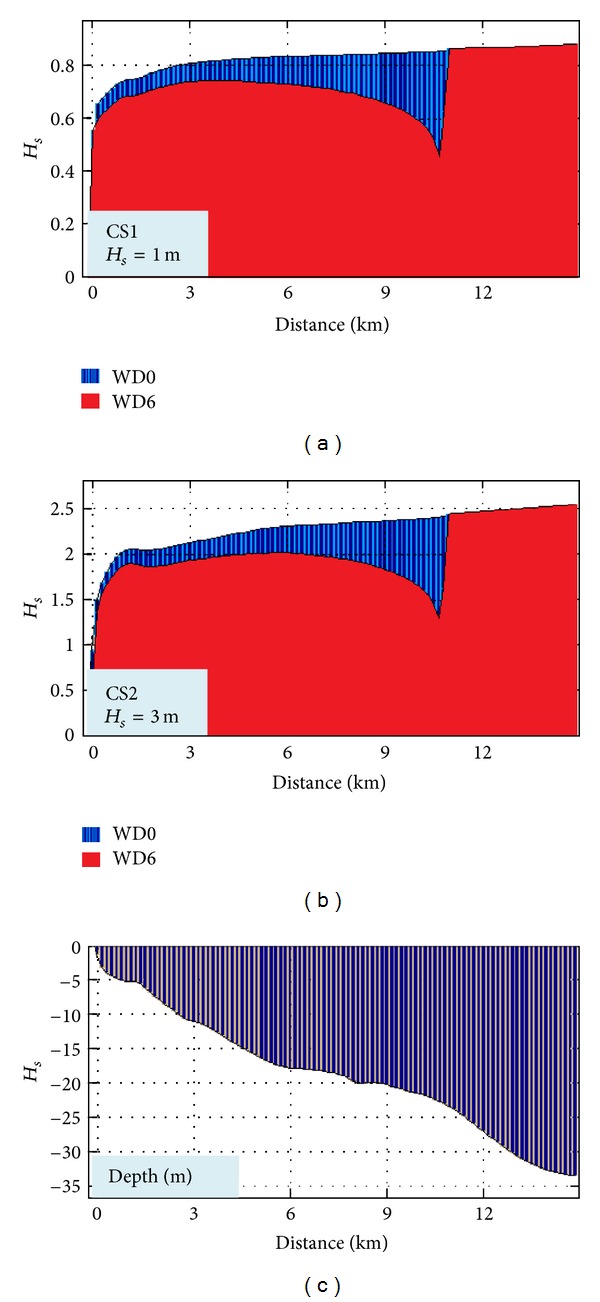
*H*_*s*_ variation along the reference line 1 without and with WD farm (WD0, WD6) for the two cases considered (CS1, CS2) and the variation of the water depth along the reference line.

**Figure 13:**
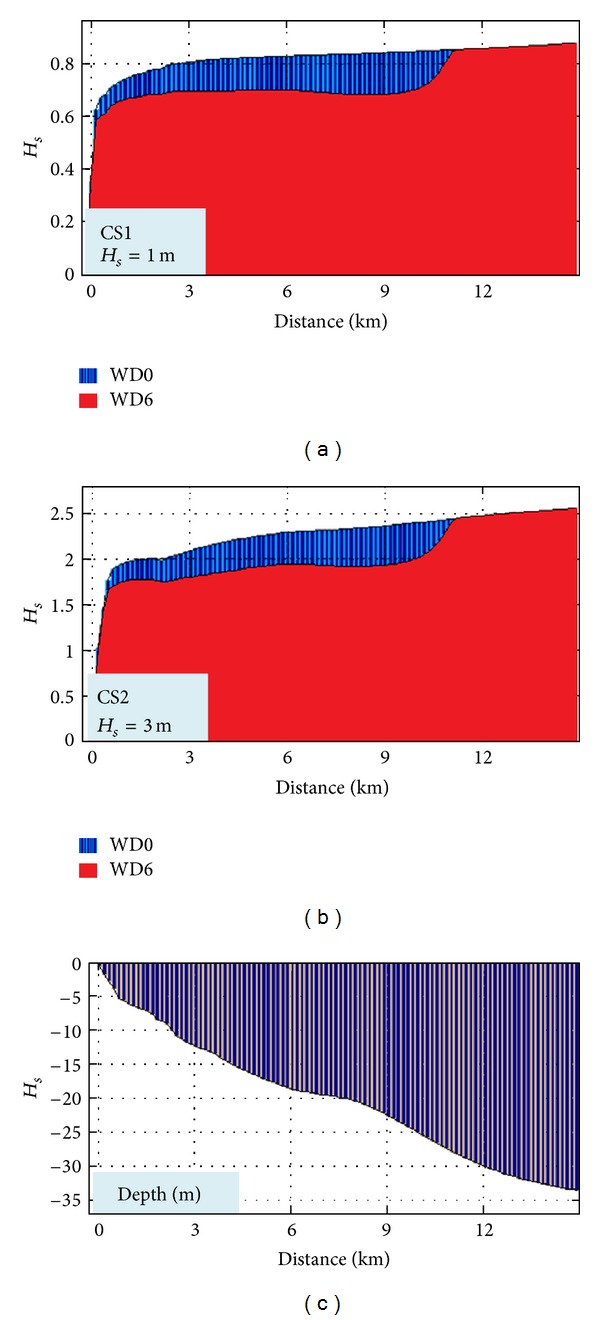
*H*_*s*_ variation along the reference line 2 without and with WD farm (WD0, WD6) for the two cases considered (CS1, CS2) and the variation of the water depth along the reference line.

**Figure 14:**
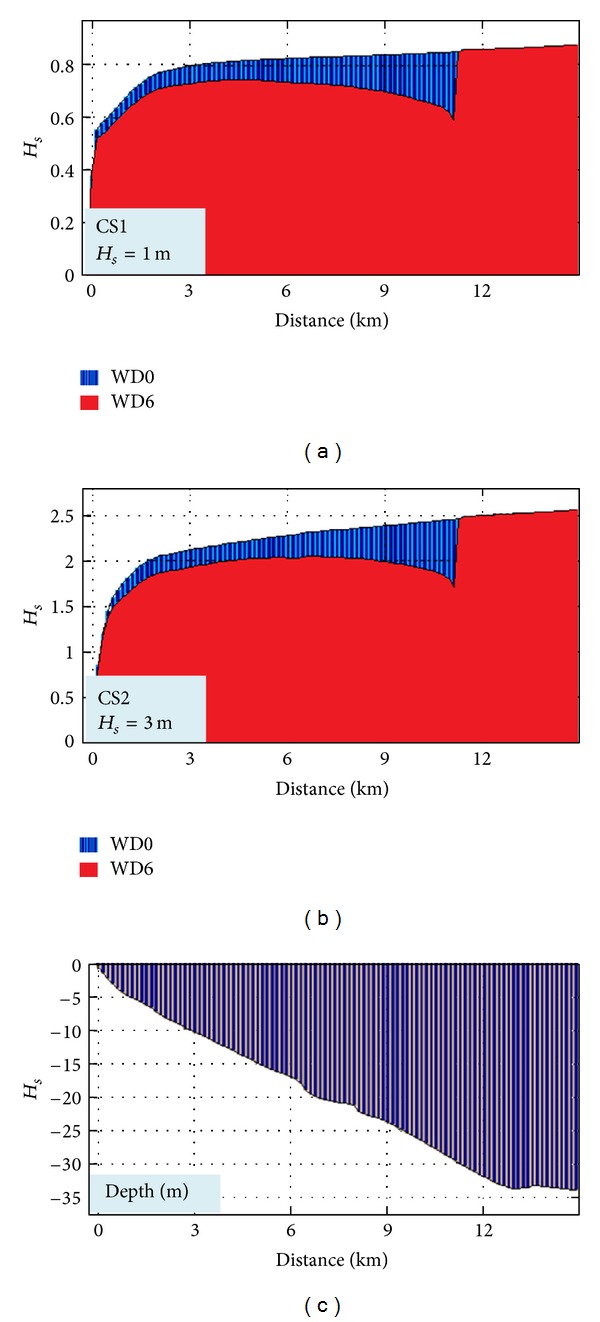
*H*_*s*_ variation along the reference line 3 without and with WD farm (WD0, WD6) for the two cases considered (CS1, CS2) and the variation of the water depth along the reference line.

The results are presented in Figure 12 (for line 1), Figure 13 (for line 2), and Figure 14 (for line 3). They all present the evolution of the waves for the two situations WD0 (blue) and WD6 (red). The bathymetric variation along the reference lines is also illustrated in each figure. As it can be seen, the most relevant impact occurs at the reference line 1 in both cases (CS1, CS2), and the lowest is at the reference line 2 due to the fact that the line is passing between two devices while in the other two cases the lines pass directly through the body of one WD.

**Figure 15:**
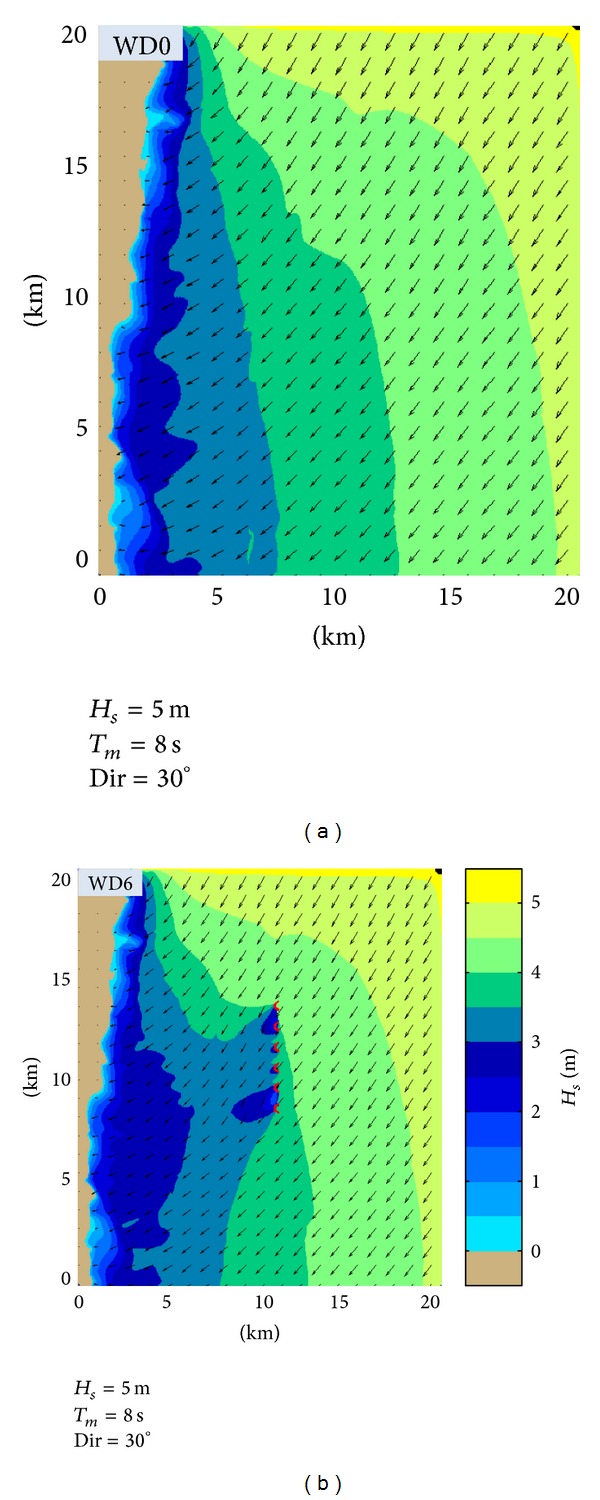
*H*_*s*_ variation along the reference line 3 without and with WD farm (WD0, WD6) for the two cases considered (CS1, CS2) and the variation of the water depth along the reference line.

**Figure 16:**
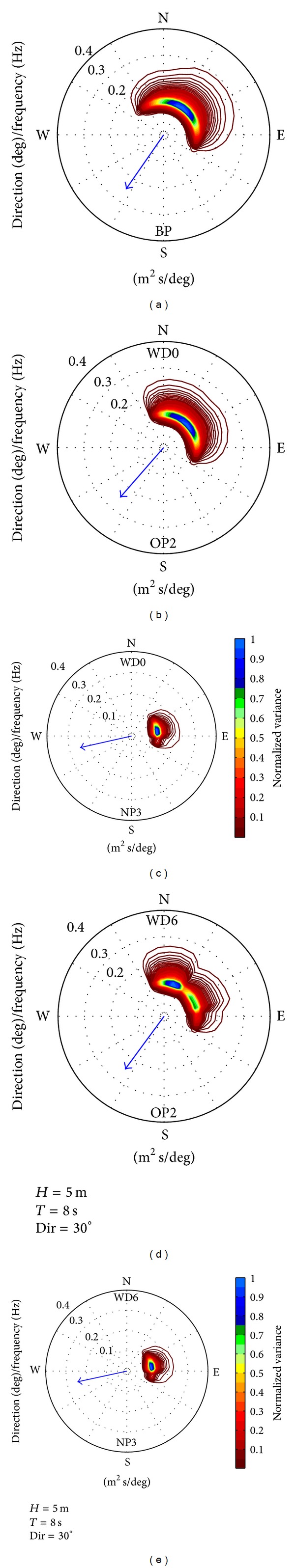
Evaluation in the spectral space of the impact on the wave field of a wave farm based on Wave Dragon WECs that operate in the target area for an additional case study defined by the parameters *H*_*s*_ = 5 m, *T*_*m*_ = 8 s, Dir = 30°. (a) BP for WD0. (b) OP2 for WD0. (c) NP3 for WD0. (d) OP2 for WD6. (e) NP3 for WD6.

**Figure 17:**
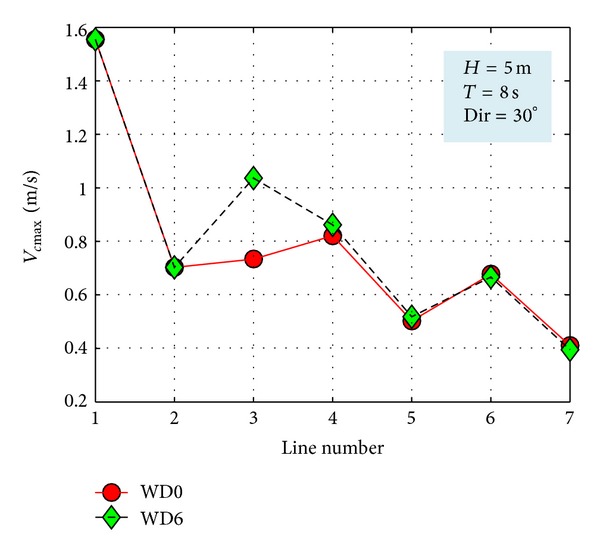
Evaluation of the impact of the energy farms on the maximum velocities of the nearshore currents along the reference lines considered for an additional case study defined by the parameters *H*_*s*_ = 5 m, *T*_*m*_ = 8 s, and Dir = 30°.

**Figure 18:**
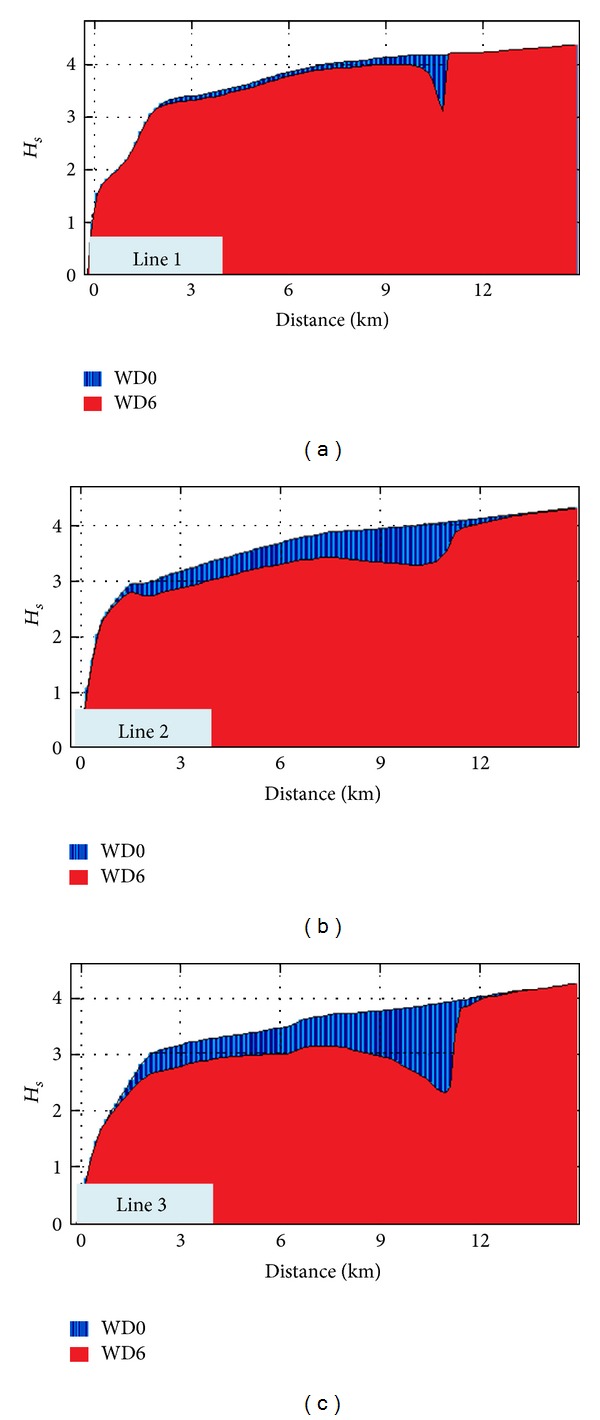
*H*_*s*_ variation along the three reference lines without and with the WEC array (WD0 and WD6) for the wave conditions corresponding to the parameters: *H*_*s*_ = 5 m, *T*_*m*_ = 8 s, and Dir = 30°.

Finally, in order to complete the picture, another case study that was analyzed will be presented. It considers the following conditions on the external boundaries: *H*_*s*_ = 5 m, *T*_*m*_ = 8 s, and Dir = 30°. Thus, Figure 15 illustrates the impact in the geographical space on the wave field and Figure 16 the evaluation in the spectral space of the impact on the wave field of the Wave Dragon farm. In this case study, the maximum values of the velocities of the nearshore currents along the reference lines are illustrated in Figure 17. In such situation, the results of the modelling system indicate that the presence of the energy farm leads this time to an increase of the nearshore currents in most places. Finally, Figure 18 presents the *H*_*s*_ variation along the three reference lines previously considered, for the two different situations without and with the WEC array.

## 6. Concluding Remarks

According to the EU requirements, 20% of the electric energy produced in Europe should be provided until 2020 by renewable energy sources. In this connection, the marine environment represents a vast space depositing a huge amount of renewable energy. Nevertheless, the most important problem related to harvesting the energy in the marine environment is represented by the high cost of the electric power produced. As regards the wave energy extraction, the most significant step in the direction of reducing the energy cost is represented by the implementation of large WEC arrays. Thus, large scale WEC deployments are expected in the near future, and a very important issue related to this perspective is to evaluate correctly the possible coastal impact of these new power plants operating in the nearshore. In this context, the present work presents an evaluation of the changes induced in the coastal wave climate by an array of six Wave Dragons. The target area considered is located in the western side of the Black Sea, but the methodology can be easily extended to any coastal environment. 

As regards the wave transformation, the modelling system considered in these evaluations is based on the SWAN spectral model, which represents an adequate framework for accounting the wave changes due to the presence of the energy farm. Evaluations were carried out in both geographical and spectral spaces for various relevant wave patterns. The results show that while immediately after the farm drastic changes occur in the wave fields, thus gradually attenuate towards the coast. In order to assess better the changes taking place in the spectral shapes due to the energy farm, transformations of theoretical JONSWAP spectra were followed for each case study considered. The results usually show that the single-peaked wave spectra are usually changed by the wave farm to double-peaked spectra immediately down wave the farm, but the spectra become again single-peaked at the level of the breaking line. This is also due to the relatively large distance between the shoreline and the location of the wave farm.

In order to assess better the changes at the level of the shoreline dynamics, the modelling system ISSM that joins SWAN with the 1D surf models was considered. This allowed an evaluation of the longshore currents. The results show that although the nearshore waves are not very much affected by the presence of the WD farm, the maximum current velocities may, however, have significant variations. These variations are most evident at the central nearshore points. The results show also that the longshore current velocity is a more sensitive parameter to the presence of the energy farm than the significant wave height. 

Since in general the presence of the energy farm has led to slight decreases of the wave conditions, its influence at the level of the shoreline dynamics is expected to be rather positive. Nevertheless, a very interesting result coming from the present work is that sometimes the presence of the energy farm may lead locally to enhancements of the longshore current velocity which means that due to the specific features of the site some coastal processes might be also accentuated. The work is still ongoing and larger WEC arrays, both of one and two lines, are being considered, which means that more accentuated changes might be expected for such configurations.

## Figures and Tables

**Table 1:** Characteristics of the computational domain defined for the SWAN simulations and the physical parameterizations activated.

SWAN model	Coordinates		Δ*x* × Δ*y* (m)		Δ*θ* (°)	Mode/scheme	*n*_*f*_	*n*_*θ*_	*n*_*gx*_ × *n*_*gy*_ = *n*_*p*_			
	Cartesian		50 × 50		5	stat/BSBT	34	35	355 × 406 = 144130			
Input/process	wave	wind	tide	crt	gen	wcap	quad	triad	diffr	bfric	setup	br
SWAN	X	X	0	X	X	0	X	X	X	X	X	X

**Table 2:** CS1 (*H*_*s*_ = 1 m, *T*_*m*_ = 4 s, Dir = 90°), evaluation of the impact of the energy farms on the waves in the reference points OP1 (northern offshore point), OP2 (central offshore point), OP3 (southern offshore point), and in the point NP1–NP7. WD0: no energy converter, WD6: four Wave Dragon energy converters operating in line.

	WD	*H*_*s*_ (m)	*E*_max⁡_ (m^2^/Hz/deg)	Dir (deg)	DSPR (deg)	*T*_*m*_/*T*_*p*_ (s)	Wlen (m)	*P*_*x*_ (m^3^/s)	*P*_*y*_ (m^3^/s)	*F*_*x*_ (N/m^2^)	*F*_*y*_ (N/m^2^)
BP	0	0.9	0.40	90.0	32.48	3.5/4	18.5	−0.13	0.00	−0.01	−0.00
	6	0.9	0.40	90.0	33.18	3.5/4	18.5	−0.13	−0.00	−0.01	−0.00
OP1	0	0.8	0.35	89.6	33.25	3.7/4	20.7	−0.10	−0.00	−0.00	−0.00
	6	0.7	0.32	91.4	33.57	3.7/4	20.4	−0.07	0.00	−0.00	0.00
OP2	0	0.8	0.31	90.0	33.23	3.7/4	20.7	−0.10	0.00	−0.00	−0.00
	6	0.7	0.31	89.3	33.81	3.7/4	20.5	−0.07	−0.00	−0.00	−0.00
OP3	0	0.8	0.35	90.4	33.23	3.7/4	20.7	−0.10	0.00	−0.00	−0.00
	6	0.6	0.30	93.1	38.28	3.7/4	20.6	−0.06	0.00	−0.00	0.00
NP1	0	0.8	0.34	80.4	30.00	3.5/4	17.1	−0.11	−0.02	0.13	0.03
	6	0.8	0.50	78.6	29.04	3.5/4	16.9	−0.11	−0.02	0.13	0.03
NP2	0	0.7	0.31	89.3	25.78	3.6/4	17.9	−0.09	−0.00	0.16	0.04
	6	0.6	0.32	86.2	26.05	3.6/4	17.6	−0.08	−0.00	0.14	0.03
NP3	0	0.7	0.34	98.8	25.54	3.5/4	15.1	−0.10	0.01	0.07	0.23
	6	0.7	0.34	99.8	24.95	3.5/4	15.0	−0.09	0.01	0.07	0.22
NP4	0	0.7	0.33	89.8	25.90	3.6/4	17.1	−0.09	−0.00	0.22	0.04
	6	0.6	0.28	90.3	27.85	3.6/4	16.8	−0.08	0.00	0.19	0.03
NP5	0	0.7	0.29	95.3	25.44	3.6/4	17.8	−0.08	0.01	0.14	−0.01
	6	0.6	0.29	98.3	26.13	3.6/4	17.5	−0.07	0.01	0.13	−0.00
NP6	0	0.7	0.29	85.4	25.90	3.6/4	17.3	−0.08	−0.01	−0.01	0.01
	6	0.6	0.29	87.3	25.60	3.6/4	17.1	−0.08	−0.01	−0.01	0.01
NP7	0	0.7	0.34	98.8	25.54	3.5/4	15.1	−0.10	0.01	0.07	0.23
	6	0.7	0.34	99.8	24.95	3.4/4	15.0	−0.09	0.01	0.07	0.22

**Table 3:** CS2 (*H*_*s*_ = 3 m, *T*_*m*_ = 6 s, and Dir = 90°), evaluation of the impact of the energy farms on the waves in the reference points OP1, OP2, OP3, and NP1–NP7.

	WD	*H*_*s*_ (m)	*E*_max⁡_ (m^2^/Hz/deg)	Dir (deg)	DSPR (deg)	*T*_*m*_/*T*_*p*_ (s)	Wlen (m)	*P*_*x*_ (m^3^/s)	*P*_*y*_ (m^3^/s)	*F*_*x*_ (N/m^2^)	*F*_*y*_ (N/m^2^)
BP	0	2.7	5.27	90.0	32.28	5.4/6	42.7	−1.74	0.00	−0.10	−0.00
	6	2.7	5.27	90.0	32.94	5.4/6	42.7	−1.73	0.00	−0.10	−0.00
OP1	0	2.3	4.31	90.5	32.44	5.6/6	46.2	−1.38	0.02	0.04	−0.01
	6	1.9	3.81	92.2	32.67	5.5/6	45.6	−0.88	0.04	0.04	0.01
OP2	0	2.4	4.31	91.0	32.39	5.6/6	46.4	−1.38	0.03	0.03	−0.01
	6	1.9	3.70	90.3	33.00	5.5/6	45.8	−0.87	0.01	0.03	−0.00
OP3	0	2.4	4.32	91.5	32.43	5.6/6	46.5	−1.38	0.04	0.02	−0.03
	6	1.8	3.64	94.3	37.56	5.6/6	46.1	−0.74	0.06	0.04	−0.02
NP1	0	2.2	4.92	78.9	26.10	5.5/6	33.6	−1.31	−0.25	−0.77	−0.60
	6	2.2	5.02	77.2	25.23	5.5/6	33.4	−1.29	−0.29	−0.64	−0.55
NP2	0	1.8	4.55	89.2	19.58	5.6/6	33.1	−0.96	−0.01	0.06	0.23
	6	1.7	4.60	86.5	19.64	5.6/6	32.9	−0.85	−0.05	0.50	0.30
NP3	0	1.5	3.08	100.1	20.07	5.4/6	28.4	−0.56	0.10	−1.48	0.45
	6	1.5	3.10	100.4	19.81	5.4/6	28.4	−0.56	0.10	−1.47	0.46
NP4	0	1.6	3.90	93.9	18.68	5.6/6	29.4	−0.69	0.04	−3.26	−0.05
	6	1.5	3.15	93.8	20.22	5.6/6	29.2	−0.64	0.04	−2.54	−0.08
NP5	0	1.7	3.46	95.0	19.98	5.6/6	31.6	−0.79	0.06	−0.24	−0.14
	6	1.6	3.51	96.9	20.23	5.5/6	31.4	−0.72	0.08	0.18	0.00
NP6	0	1.7	3.63	83.6	18.51	5.6/6	31.8	−0.79	−0.10	−0.98	−0.31
	6	1.6	3.74	84.5	18.20	5.6/6	31.7	−0.78	−0.08	−0.89	−0.25
NP7	0	1.5	3.08	100.1	20.07	5.4/6	28.4	−0.56	0.10	−1.48	0.45
	6	1.5	3.10	100.4	19.81	5.4/6	28.4	−0.56	0.10	−1.47	0.46

**Table 4:** Evaluation of the impact of the energy farms on the waves in the reference points OP1, OP2, and OP3 for the wave conditions (a) *H*_*s*_ = 1 m, *T*_*m*_ = 4 s, and Dir = 30° and (b) *H*_*s*_ = 1 m, *T*_*m*_ = 4 s, and Dir = 150°.

	*N*	*H*_*s*_ (m)	*E*_max⁡_ (m^2^/Hz/deg)	Dir (deg)	DSPR (deg)	*T*_*m*_/*T*_*p*_ (s)	Wlen (m)	*P*_*x*_ (m^3^/s)	*P*_*y*_ (m^3^/s)	*F*_*x*_ (N/m^2^)	*F*_*y*_ (N/m^2^)
(a) Direction: 30°											

BP	0	0.8	0.39	34.1	32.76	3.6/4	19.1	−0.06	−0.10	−0.00	−0.01
	6	0.8	0.40	33.9	33.10	3.6/4	19.1	−0.06	−0.10	−0.00	−0.01
OP1	0	0.8	0.35	32.2	31.52	3.7/4	20.4	−0.05	−0.09	−0.00	−0.00
	6	0.7	0.36	24.1	31.75	3.6/4	20.1	−0.03	−0.07	−0.00	−0.00
OP2	0	0.8	0.35	33.1	31.29	3.7/4	20.5	−0.05	−0.08	−0.00	−0.00
	6	0.7	0.35	27.2	32.50	3.6/4	20.2	−0.03	−0.07	−0.00	−0.00
OP3	0	0.8	0.34	34.2	30.93	3.7/4	20.6	−0.05	−0.08	−0.00	−0.00
	6	0.7	0.34	26.4	30.07	3.6/4	20.3	−0.03	−0.06	−0.00	−0.00

(b) Direction: 150°											

OP1	0	0.8	0.34	146.3	31.50	3.7/4	20.7	−0.05	0.08	−0.00	0.00
	6	0.7	0.33	152.2	32.59	3.7/4	20.4	−0.03	0.07	−0.00	0.00
OP2	0	0.8	0.34	147.1	31.70	3.7/4	20.6	−0.05	0.08	−0.00	0.00
	6	0.7	0.35	155.3	31.63	3.7/4	20.5	−0.03	0.07	0.00	0.00
OP3	0	0.8	0.35	147.9	31.95	3.7/4	20.5	−0.05	0.09	−0.00	0.00
	6	0.7	0.35	157.1	27.94	3.7/4	20.5	−0.03	0.08	−0.00	0.00

**Table 5:** Evaluation of the impact of the energy farms on the waves in the reference points OP1, OP2, and OP3 for the wave conditions (a) *H*_*s*_ = 3 m, *T*_*m*_ = 6 s, and Dir = 30° and (b) *H*_*s*_ = 3 m, *T*_*m*_ = 6 s, and Dir = 150°.

	*N*	*H*_*s*_ (m)	*E*_max⁡_ (m^2^/Hz/deg)	Dir (deg)	DSPR (deg)	*T*_*m*_/*T*_*p*_ (s)	Wlen (m)	*P*_*x*_ (m^3^/s)	*P*_*y*_ (m^3^/s)	*F*_*x*_ (N/m^2^)	*F*_*y*_ (N/m^2^)
(a) Direction: 30°											

BP	0	2.6	5.12	35.0	32.51	5.4/5.8	43.8	−0.90	−1.30	−0.04	−0.05
	6	2.6	5.12	34.9	32.84	5.4/5.8	43.9	−0.90	−1.30	−0.04	−0.05
OP1	0	2.3	4.24	34.8	30.62	5.5/5.8	45.9	−0.70	−1.10	0.04	−0.04
	6	2.0	4.31	27.0	31.07	5.5/5.8	45.1	−0.40	−0.90	0.05	−0.02
OP2	0	2.2	4.17	36.0	30.24	5.5/5.8	46.0	−0.70	−1.00	0.03	−0.03
	6	1.9	4.12	30.6	31.61	5.5/5.8	45.2	−0.50	−0.80	0.04	−0.01
OP3	0	2.2	4.11	37.2	29.97	5.5/5.8	46.2	−0.70	−1.00	0.03	−0.05
	6	1.9	4.02	29.8	29.15	5.5/5.8	45.4	−0.40	−0.80	0.03	−0.03

(b) Direction: 150°											

OP1	0	2.2	4.01	143.4	30.12	5.5/5.8	46.1	−0.70	1.00	0.04	0.01
	6	1.9	3.95	148.8	31.38	5.4/5.8	45.5	−0.50	0.80	0.04	0.01
OP2	0	2.2	4.10	144.4	30.49	5.5/5.8	46.1	−0.70	1.00	0.03	0.02
	6	2.0	4.14	152.3	30.67	5.4/5.8	45.6	−0.40	0.80	0.05	0.01
OP3	0	2.3	4.21	145.7	30.79	5.5/5.8	46.0	−0.70	1.10	0.03	0.01
	6	2.0	4.23	154.9	26.79	5.4/5.8	45.9	−0.40	1.00	0.04	0.01

**Table 6:** Evaluation of the impact of the energy farms on the waves in the reference points OP1, OP2, and OP3 for the wave conditions (a) *H*_*s*_ = 5 m, *T*_*m*_ = 8 s, and Dir = 30°, (b) *H*_*s*_ = 5 m, *T*_*m*_ = 8 s, and Dir = 90°, and (c) *H*_*s*_ = 5 m, *T*_*m*_ = 8 s, and Dir = 150°.

	*N*	*H*_*s*_ (m)	*E*_max⁡_ (m^2^/Hz/deg)	Dir (deg)	DSPR (deg)	*T*_*m*_/*T*_*p*_ (s)	Wlen (m)	*P*_*x*_ (m^3^/s)	*P*_*y*_ (m^3^/s)	*F*_*x*_ (N/m^2^)	*F*_*y*_ (N/m^2^)
(a) Direction: 30°											

BP	0	4.5	18.51	34.6	32.15	7.1/8.2	72.9	−3.70	−5.50	−0.08	−0.17
	6	4.5	18.51	34.4	32.52	7.1/8.2	73.0	−3.70	−5.50	−0.08	−0.17
OP1	0	3.9	15.12	39.9	29.34	7.2/8.2	73.2	−3.70	−4.30	0.43	−0.24
	6	3.3	13.65	32.3	30.60	7.2/8.2	72.0	−2.20	−3.40	0.43	−0.10
OP2	0	3.8	15.07	41.0	28.81	7.2/8.2	73.1	−3.60	−4.00	0.36	−0.16
	6	3.3	12.46	36.2	30.66	7.2/8.2	72.1	−2.30	−3.10	0.32	−0.07
OP3	0	3.8	14.97	42.5	28.63	7.2/8.2	73.4	−3.60	−3.80	0.30	−0.27
	6	3.1	11.93	35.5	28.12	7.2/8.2	72.3	−2.10	−2.90	0.25	−0.28

(b) Direction: 90°											

OP1	0	3.9	16.90	92.7	30.22	7.2/8.2	73	−5.80	0.30	0.40	−0.06
	6	3.1	14.48	94.1	29.96	7.2/8.2	72.3	−3.70	0.20	0.20	0.14
OP2	0	4	16.68	93.1	30.14	7.2/8.2	73.5	−5.90	0.30	0.30	−0.03
	6	3.2	14.02	91.8	30.66	7.2/8.2	72.8	−3.70	0.10	0.20	0.02
OP3	0	4	16.40	93.6	30.30	7.2/8.2	74.1	−5.90	0.40	0.30	−0.10
	6	3.0	13.42	96.1	35.52	7.2/8.2	73.3	−3.10	0.30	0.30	−0.10

(c) Direction: 150°											

OP1	0	3.8	15.29	139.9	27.89	7.2/8.2	72.9	−3.50	4.10	0.39	0.06
	6	3.3	13.96	144.5	29.51	7.2/8.2	71.7	−2.30	3.20	0.37	0.08
OP2	0	3.8	15.57	140.9	28.56	7.2/8.2	73.1	−3.50	4.30	0.36	0.05
	6	3.3	15.61	148.3	29.19	7.2/8.2	72.1	−2.20	3.50	0.40	0.08
OP3	0	3.9	15.87	142.5	28.78	7.2/8.2	73.3	−3.50	4.50	0.34	−0.10
	6	3.5	15.90	151.3	25.04	7.2/8.2	72.9	−2.30	4.10	0.42	0.10

**Table 7:** Evaluation of the impact of the energy farms on the nearshore currents in terms of maximum current velocities along the reference lines RL1–RL7 for *H*_*s*_ = 1 m, *H*_*s*_ = 3 m, *H*_*s*_ = 5 m, and three different wave directions (30°, 90°, 150°). The two configurations (WD0 and WD6) were considered in parallel.

Case study	Line	L1	L2	L3	L4	L5	L6	L7
	config.							
H1D30	WD0	0.93	0.29	0.74	0.33	0.50	0.31	0.49
	WD6	1.16	0.40	0.75	0.33	0.53	0.30	0.48
H1D90	WD0	0.29	0.13	0.23	0.02	0.19	0.08	0.23
	WD6	0.32	0.15	0.05	0.04	0.25	0.03	0.24
H1D150	WD0	0.76	0.25	0.99	0.39	0.74	0.30	0.89
	WD6	0.73	0.24	0.97	0.38	0.74	0.30	0.89

H3D30	WD0	1.63	0.75	1.20	0.58	0.62	0.69	0.49
	WD6	1.63	0.75	1.28	0.63	0.64	1.66	0.48
H3D90	WD0	0.55	0.31	0.72	0.10	0.22	0.07	0.29
	WD6	0.68	0.33	0.49	0.21	0.26	0.05	0.30
H3D150	WD0	1.04	0.28	1.92	0.74	0.91	0.71	0.94
	WD6	1.01	0.26	1.89	0.76	0.93	0.36	0.94

H5D30	WD0	1.55	0.70	0.73	0.82	0.50	0.68	0.41
	WD6	1.55	0.70	1.04	0.86	0.52	0.67	0.40
H5D90	WD0	0.34	0.09	1.33	0.50	0.38	0.26	0.43
	WD6	0.41	0.15	1.25	0.53	0.40	0.26	0.43
H5D150	WD0	0.85	0.26	1.98	1.02	0.77	0.32	1.04
	WD6	0.82	0.24	2.04	1.14	0.77	0.32	1.04
